# A Case of Isolated Pulmonary Mycobacterium Avium Complex Being the First Presentation of a Newly Diagnosed HIV/AIDS

**DOI:** 10.7759/cureus.9223

**Published:** 2020-07-16

**Authors:** Fuad I Abaleka, Bisrat Nigussie, Ozlem Onal, Rana Al-Zakhari, Esmael Yimer

**Affiliations:** 1 Internal Medicine, Richmond University Medical Center, Staten Island, USA

**Keywords:** respiratory tract infection, immunocompromised, hiv, aids, mac, non-tuberculous mycobacteria

## Abstract

We report a case of a 39-year-old HIV positive transgender female with isolated manifestations of pulmonary Mycobacterium avium complex (MAC) infection. Although MAC infection is common in immunocompromised patients, the classical presentation is extra-pulmonary. Pulmonary MAC infection is extremely rare. The majority of cases involve patients with underlying structural lung disease. There are no case reports of isolated pulmonary MAC in HIV/AIDS patients without any structural lung disease in the last 17 years. Also, we have not found any cases of newly diagnosed HIV/AIDS patients with pulmonary MAC being the initial presentation.

## Introduction

Mycobacterium avium complex (MAC) is a free-living bacterium found in water, soil, and dust. The main route of transmission is through inhalation or ingestion and the majority of these infections involve extrapulmonary sites [1]. Most patients present with nonspecific symptoms such as fever, night sweats, weight loss, fatigue, abdominal pain, diarrhea, or focal lymphadenitis [2]. Laboratory work may show anemia, elevated alkaline phosphatase, and lactate dehydrogenase [3]. Diagnosis is made with cultures and radiographic imaging. Treatment includes a combination of antimicrobial therapy. This case reported herein describes an HIV/AIDS patient who was found to have isolated pulmonary disease caused by Mycobacterium avium complex.

## Case presentation

A 39-year-old, transgender female with no significant past medical history presented to the hospital with complaints of a persistent cough of a four-month duration. The patient stated the cough was productive with whitish sputum and was associated with weight loss, night sweats, loss of appetite, and low-grade fever. Three weeks prior to the hospital admission she had visited the emergency room with similar complaint and was discharged with oral antibiotics for community acquired pneumonia. However, symptoms persisted despite completion of antibiotics prompting a second visit to the hospital. During the second visit, the patient was admitted for further evaluation. On initial presentation, the patient was febrile - Temperature 100.4 F, HR 106 bpm, with blood pressure of 113/71 mmHg and an O2 saturation of 96% on room air. The patient was not in acute distress, was alert awake and oriented times three. Physical exam was notable for pink conjunctiva, non-icteric sclera, no oropharyngeal lesions, no lymphadenopathy; chest examination revealed good air entry bilaterally with diffuse crackles worse on right upper posterior lung, resonance to percussion; Cardiovascularly: S1 and S2 heard, no murmur or gallops; Abdomen: normal bowel sounds, soft, non-tender, non-distended and no organomegaly.

**Figure 1:**
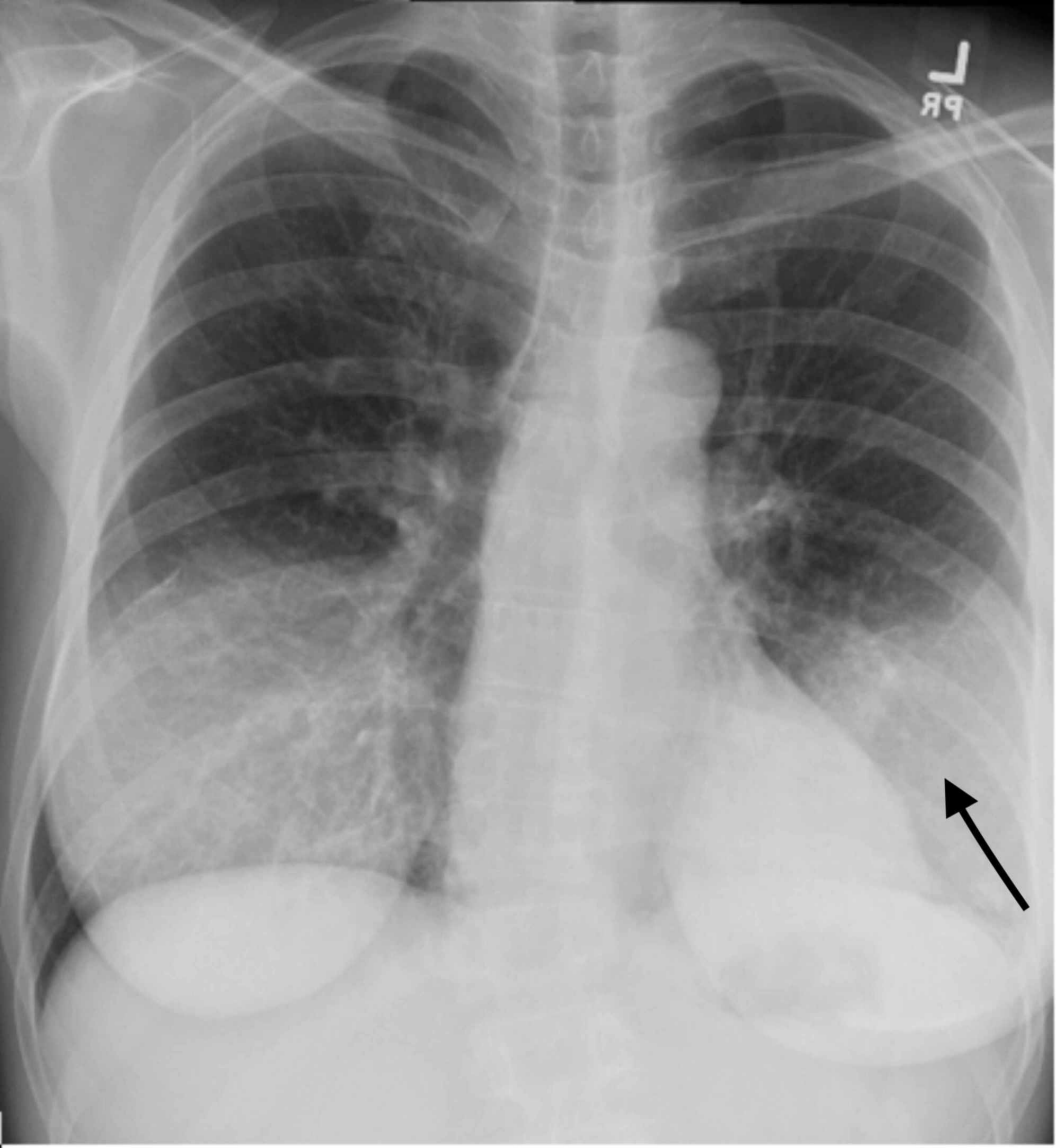
Chest X-ray with an arrow showing left lower lobe consolidation, bilateral reticular and interstitial markings.

**Figure 2:**
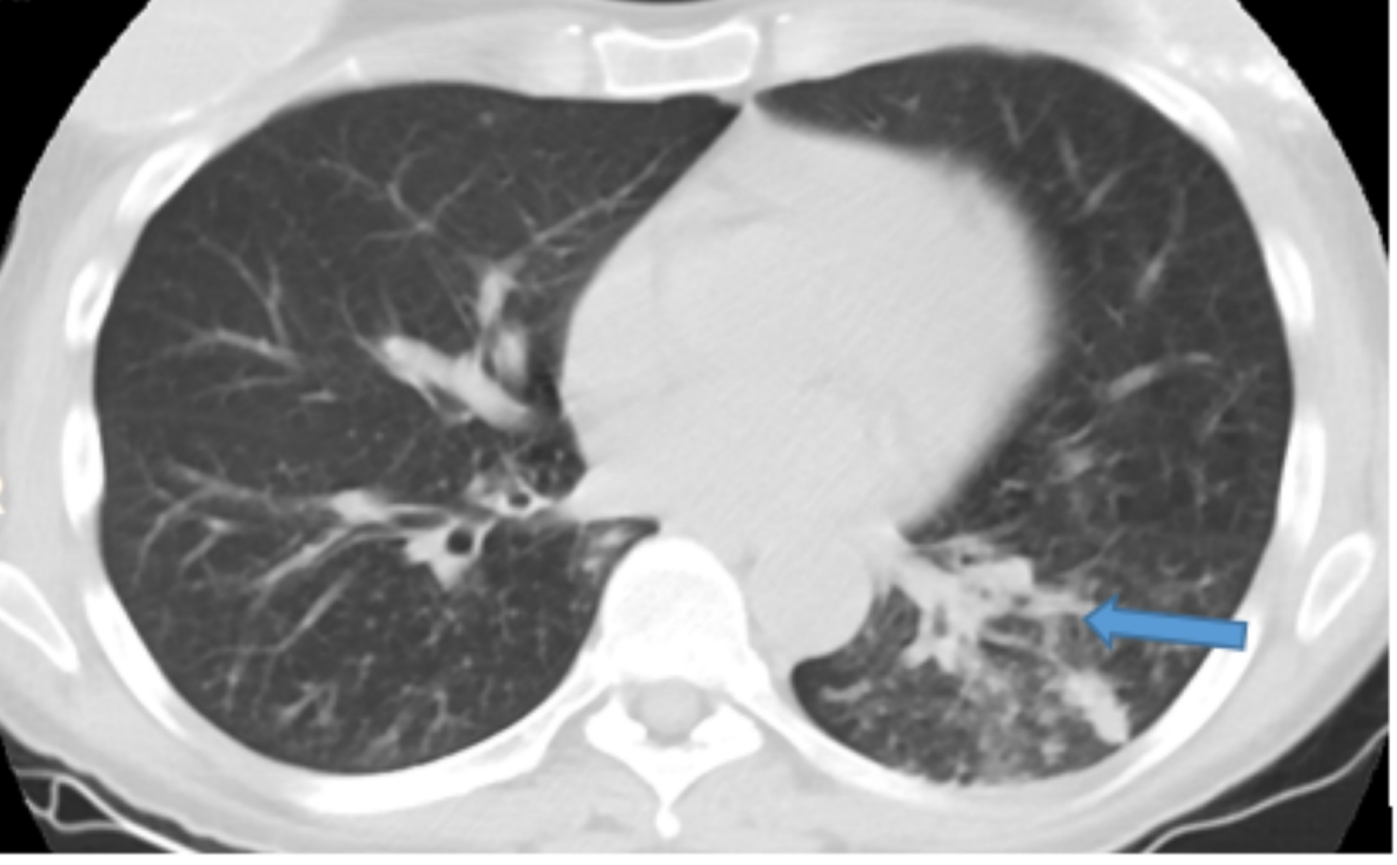
CT chest showing bilateral infiltration and ground glass opacification. The blue arrow is showing a tree-in-bud appearance with the nodular opacity in the left lower lobe.

Given the above history and physical examination, the patient was being evaluated for HIV and pulmonary TB. Labs showed WBC 13 k/ul, hemoglobin 11 g/dL, platelets 574 k/ul, elevated erythrocyte sedimentation rate (ESR) 110, lactate dehydrogenase (LDH) 193 U/L, chemistry is within normal limit, HIV test positive, CD4 count of 73 cells/mcL, viral load 559,000 copies/mL, sputum acid fast bacilli (AFB) negative x3, Grocott’s methenamine silver stain for Pneumocystis jiroveci pneumonia (PJP) was negative. Chest X-ray (Figure 1) revealed left lower lobe, patchy to confluent opacity with air bronchograms. CT chest showed left lower lobe consolidation and tree-in-bud appearance with the nodular opacity as seen in Figure 2.

During hospitalization, the patient was being treated for community-acquired pneumonia with ceftriaxone and azithromycin. All of her tests for pulmonary tuberculosis were negative and she was discharged home after mild symptomatic improvement. Prior to discharge, the patient was started on PJP prophylaxis and highly active antiretroviral therapy (HAART) for HIV positive patient with AIDS. On subsequent visit to the infectious disease clinic, she continued to have persistent cough with other constitutional symptoms. Physician revealed that her respiratory culture was positive of MAC identified by DNA probe. The patient was prescribed standard MAC treatment regimen: azithromycin 500 mg daily, rifampin 600 mg daily, and ethambutol 25 mg/kg/day for a duration of 12 months. On subsequent follow-up in infectious disease clinic, she showed marked improvement of her symptoms.

## Discussion

This is a young patient who came with no significant medical history of structural lung disease who presented with persistent cough of four months duration that did not respond to multiple trials of antibiotics. While at the RUMC, she was diagnosed with HIV positive with full-blown AIDS and found to have this rare disease, pulmonary MAC. MAC infection typically affects extrapulmonary sites. MAC pulmonary disease occurs in patients with known structural lung diseases such as chronic obstructive pulmonary disease and bronchiectasis [4]. Otherwise isolated pulmonary MAC infection in patients without prior structural lung disease is uncommon. In patients with HIV, an isolated pulmonary MAC infection is extremely rare, even though colonization of the lungs is common and may be predictive of disseminated disease. To our knowledge, reports of isolated pulmonary MAC infection began to appear in the medical literature in 1988, after zidovudine became available as an anti-retroviral agent. Including our patient, there are now 26 reported cases of isolated pulmonary MAC infection in HIV-infected individuals [4-12]. However, there are no reported cases of isolated pulmonary MAC over the last 17 years, with the last case report being in 2003. This is likely due to the advent of newer and highly active antiretroviral therapies.

The diagnosis of isolated pulmonary MAC is difficult as it takes weeks for non-tuberculous mycobacterium to grow on culture. In our case, the results of sputum culture were reported after 26 days. The patient was misdiagnosed as community-acquired pneumonia and treated with multiple courses of antibiotics. The number of days it took to get a culture report posed a diagnostic challenge for us. Unless physicians have a high index of suspicion, especially when there is a treatment failure in high-risk patients may be misdiagnosed. In addition, to the best of our knowledge, this case is the first case of an isolated pulmonary MAC as the initial presentation of a newly diagnosed HIV/AIDS patient. Generally, it is recommended to delay the initiation of HAART therapy in patients with opportunistic infections like MAC in order to reduce the chances of immune reconstitution syndrome, which has been reported [4-8].

## Conclusions

The diagnosis of isolated pulmonary MAC in HIV/AIDS without structural pulmonary disease has not been reported since 2003, which makes our case unique. Furthermore, there has never been a case report of newly diagnosed HIV/AIDS patient presenting in whom initial presentation was isolated pulmonary MAC.
